# Study on Remote Field Eddy Current Testing Technology for Crack-like Defects in Long Truss Structure of Aircraft

**DOI:** 10.3390/ma15155093

**Published:** 2022-07-22

**Authors:** Lipan Zhang, Rui Deng, Ning Ning, Junling Fan, Wentao Wang, Kai Song

**Affiliations:** 1 School of Aeronautical Manufacturing Engineering, Nanchang Hangkong University, Nanchang 330063, China; 30010@nchu.edu.cn (L.Z.); dengruiisme@163.com (R.D.); 2 Aircraft Strength Research Institute of China, Xi’an 710065, China; df.ning@163.com (N.N.); fanjunling@mail.dlut.edu.cn (J.F.); 3 School of Testing and Optoelectronic Engineering, Nanchang Hangkong University, Nanchang 330063, China; wentao20212021@163.com

**Keywords:** aircraft long truss structure, crack detection, remote field eddy current, finite element simulation

## Abstract

Detection of hidden defects of aircraft long truss structures (aluminum alloy) is a challenging problem. The shape of the aircraft truss structure is complex, and the crack defects are buried in a large depth. Without the restriction of skin effect, remote field eddy current (RFEC) has great advantages in detecting buried depth defects. In this paper, in order to detect the hidden defects of the aluminum alloy aircraft long truss structure, the remote field eddy current probe is improved from two aspects of magnetic field enhancement and near-field signal suppression using the finite element method. The results show that indirect coupling energy is greatly enhanced when the connected magnetic circuit is added to the excitation coil. By adding a composite shielding structure outside the excitation coil and the detection coil, respectively, the direct coupling energy is effectively restrained. As a result, the size of the probe is reduced. By optimizing the coil spacing and probe placement position, the detection sensitivity of the probe is improved. The simulation is verified by experiments, and the experimental results are consistent with the simulation conclusions.

## 1. Introduction

**Figure 1:**
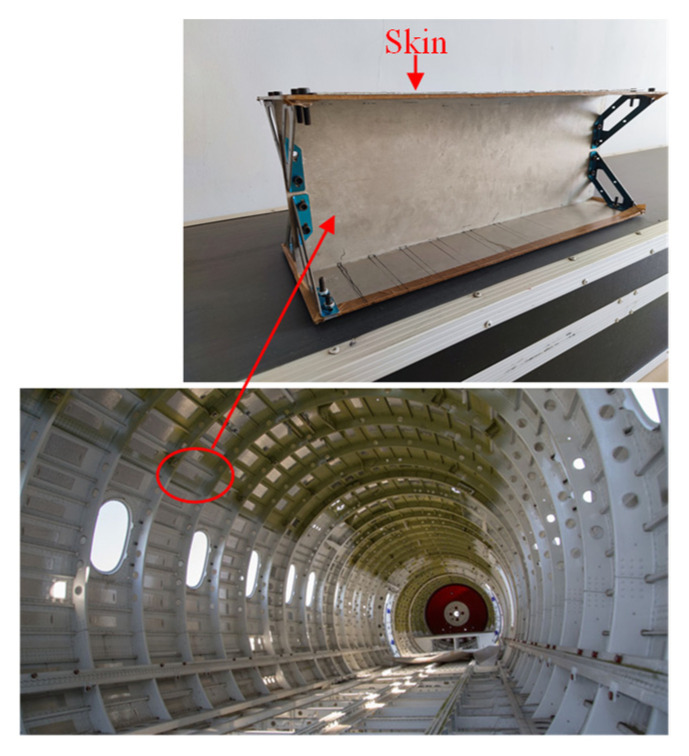
The physical figure of the aircraft long truss component.

As a high-strength aluminum alloy component bearing longitudinal force, the aircraft long truss component easily produces crack-like defects during service, resulting in the failure of components or mechanical equipment and even major accidents [1,2]. The physical figure of the aircraft long truss component is shown in Figure 1.

At present, scholars and engineering inspectors have carried out numerous scientific studies on the detection of aircraft structural defects. In [3], a method to monitor full-scale aircraft fatigue cracks using strain data was proposed. The strain gauge can pick up the changes at the crack initiation stage, but it is also sensitive to the sticking position and direction of the strain gauge. Cao [4] applied eddy current testing technology to carry out the detection of six and seven ribs of the third wall of the central wing of a certain type of aircraft. However, the buried depth of the crack-like defects of the aircraft long truss components exceeded the detection range of low-frequency eddy current testing, making it difficult to detect. Wang [5] applied acoustic emission monitoring technology to test and study the fatigue fracture of aircraft metal riveted parts. She successfully monitored the generation of fatigue cracks in riveted structures and obtained important parameters of fatigue crack initiation. Geng [6] also used acoustic emission technology to monitor the whole process of fatigue cracks of the third-generation aircraft and found defects. They proved that acoustic emission technology could also be used to monitor the strength damage of full-scale aircraft [7].

Although scholars and engineering inspectors have carried out relevant testing and detection work, such as strain measurement and acoustic emission detection technology, there are still some problems, such as inconvenient operation and sensitive detection information to the number and position of probes. Eddy current testing technology has been applied in the detection of aircraft structure because of its characteristics of no coupling and simple operation.

For surface and near-surface defects, conventional eddy current or low-frequency eddy current testing technology can be utilized. For example, Wu [8,9] proposed a DC-biased magnetization based ECT (DCMECT) technique which can detect the subsurface defect with a buried depth up to 6 mm. While for deep buried crack-like defects, even low-frequency eddy current testing technology is difficult to effectively detect defects. Additionally, constrained by the assembly environment, the actual detection area of a long truss structure is usually narrow. Therefore, it is of great significance to develop a non-destructive testing method for rapid detection of hidden defects of aircraft long truss structures, which can prevent the occurrence of serious and malignant accidents.

RFECT (Remote field eddy current testing) technology is a new branch of eddy current testing. Its main feature is its large detection depth [10,11]. In 2011, Thirunavukkarasu [12,13] proposed an effective signal processing technology in RFECT, which can effectively detect the bending part in the generator pipe. Kobayashi [14] strengthened the excitation magnetic field by adding a magnetic circuit on the excitation coil and adopted the circumferential arrangement of multiple detection coils. The feasibility of using remote field eddy current to detect double-walled pipes with steel mesh layers was verified by experiments. In 2013, Rosado [15] proposed an efficient design method for eddy current testing probes. In 2017, Xu [10,16] designed an external probe for the pipeline to quantitatively analyze the internal and external wall defects of the pipeline elbow. In 2019, Efimov [17] used numerical simulation to study the detection effect of remote field eddy current of metal tubes, put forward its unique characteristics, pointed out the advantages and disadvantages of metal remote field eddy current testing, and submitted the probe design for remote field eddy current testing. However, the above are studies on pipeline remote field eddy current testing technology, which cannot be used to detect aircraft long truss structure. In the research of plane remote field eddy current testing (PRFECT) technology in 2018, Chang [18] designed and optimized reflective and transmissive eddy current probes and analyzed their detection mechanism. The results showed that the reflective probe could only detect the surface defects of the flat plate, while the transmissive probe can detect the buried depth defects. In 2020, Zhang [19] proposed the use of double symmetric detection coils to eliminate false peaks in remote field eddy current testing of unidirectional carbon fiber composites. Yang [20,21,22] optimized the remote field eddy current probe using magnetic field shielding technology. Adding an excitation coil with a shielding structure can make the indirect magnetic field focus through the flat plate, reduce the diffusion of magnetic field energy along the direct coupling path, and improve the detection ability of the PRFECT probe for buried defects. However, there is also the problem of the large size of the probe, which makes it difficult to use the probe when the detection area is small.

To solve the problem of the larger probe size above, this paper uses the PRFECT method to detect the crack-like defects of aircraft long truss structures. Based on the detection principle of the PRFECT method, the influence of crack-like defects of aircraft long truss structure on remote field eddy current signal is studied in depth using finite element technology, and the remote field eddy current testing probe is improved. According to the simulation results, the remote field eddy current probe is designed and processed, which greatly reduces the volume of the remote field eddy current probe. Additionally, the crack-like defect detection test of aircraft long truss structure is carried out. This method provides useful exploration for the engineering application of remote field eddy current in the defect detection of aircraft long truss structures.

## 2. Remote Field Eddy Current Testing Principle of Aircraft Long Truss Structure

**Figure 2:**
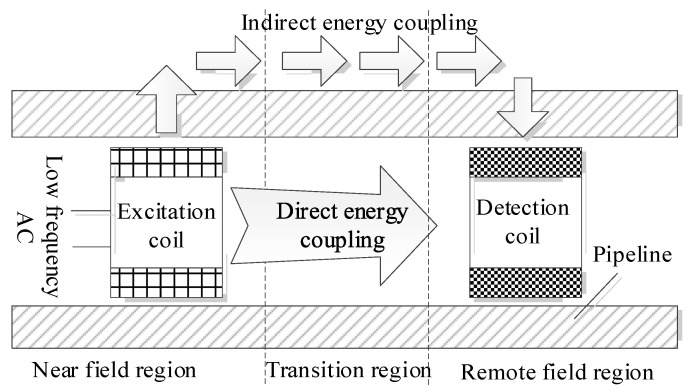
Schematic diagram of pipeline remote field eddy current testing.

The traditional remote field eddy current probe is composed of an excitation coil and a detection coil. The inner through eddy current coil is placed on the inner wall of the pipe, and the excitation coil and detection coil are placed coaxially, as shown in Figure 2. The excitation coil loads a low-frequency sinusoidal signal to generate a low-frequency excitation magnetic field. Due to the shielding effect of the pipe, the direct coupling magnetic field decays rapidly, and the indirect coupling magnetic field penetrates the pipe wall twice to reach the detection coil. The detection coil is located at 2~3 times the pipe diameter of the excitation coil to receive the remote field signal.

**Figure 3:**
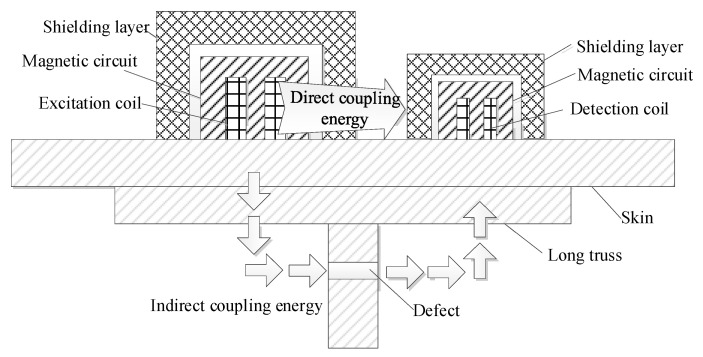
The principle of plane remote field eddy current testing of aircraft long truss structure.

Different from the pipeline structure, when detecting the aircraft long truss structure, the probe needs to be placed on the aircraft skin, and the defect is located in the web part of the T-shaped long truss structure, as shown in Figure 3. Since the long truss structure does not have the shielding effect of the pipeline, it is impossible to directly place the coil on the skin to produce the remote field eddy current effect. Therefore, it is necessary to improve the structure of the traditional remote field eddy current probe. A remote field signal enhancement unit is added to guide the propagation of the magnetic field through the magnetic circuit to enhance the indirect coupling magnetic field. A composite shielding damping structure is added to hinder the propagation of the directly coupled magnetic field so that the energy penetrates the inspected component twice through the indirect coupling channel to reach the detection coil.

## 3. Simulation Optimization of PRFECT Probe

**Figure 4:**
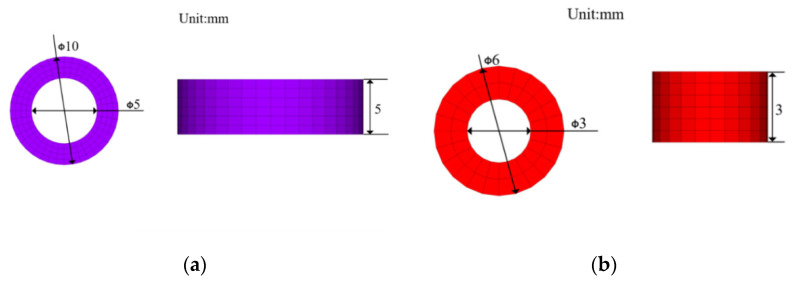
Coil size diagram. (**a**) Excitation coil; (**b**) Detection coil.

According to theoretical analysis, in order to realize the remote field eddy current effect in the long truss structure, the PRFECT probe needs to be improved from the magnetic circuit coupling unit and the shielding suppression unit. Taking the hollow cylindrical coil with the normal vertical plane as the research object, the excitation coil height is 5 mm, inner diameter is 8 mm, outer diameter is 10 mm, number of turns is 600, detection coil height is 3 mm, inner diameter is 4 mm, outer diameter is 6 mm, and the number of turns is 900, as shown in Figure 4a,b.

**Figure 5:**
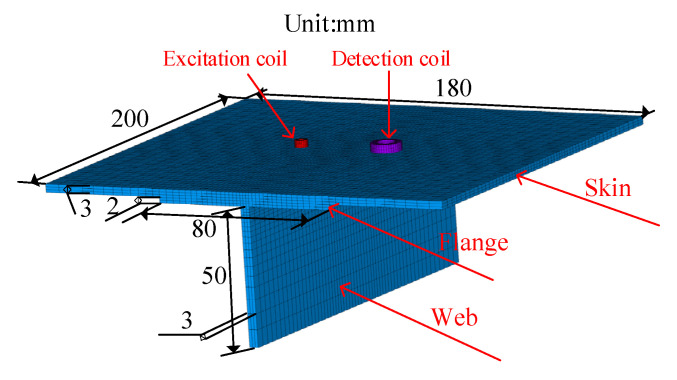
Aircraft long truss size diagram.

First, a simulation model of long truss members without defects is established. The model is mainly composed of a T-shaped long truss structure and skin. The size of the skin is 200 mm (length) × 180 mm (width) × 3 mm (thickness), the flange of the T-shaped long truss structure is 200 mm (length) × 80 mm (width) × 2 mm (thickness), and the web part is 200 mm (length) × 50 mm (width) × 3 mm (thickness), as shown in Figure 5.

The excitation frequency f is 400 Hz and drive current i is 100 mA, and the influence of magnetic circuit structure, shielding layer material, excitation frequency, and other factors on the electromagnetic field distribution is studied. Then, the simulation model of an aircraft long truss member with crack-like defects is established, and the effects of excitation/detection coil spacing and excitation coil position on the detection sensitivity of the PRFECT probe are analyzed.

### 3.1. Simulation and Optimization of Magnetic Circuit Coupling Unit

The excitation magnetic field enhancement unit is mainly composed of the magnetic circuit installed on the excitation coil. The magnetic circuit composed of materials with high permeability has a stronger ability to gather and guide magnetic field propagation. Therefore, the ferrite with high permeability is selected as the magnetic circuit material in the simulation. The structure and size of the magnetic circuit directly affect the propagation characteristics of the magnetic field, so the magnetic circuits with different structures and sizes are simulated and analyzed.

#### 3.1.1. The Influence of Magnetic Circuit Structure on Indirectly Coupled Magnetic Field

**Figure 6:**
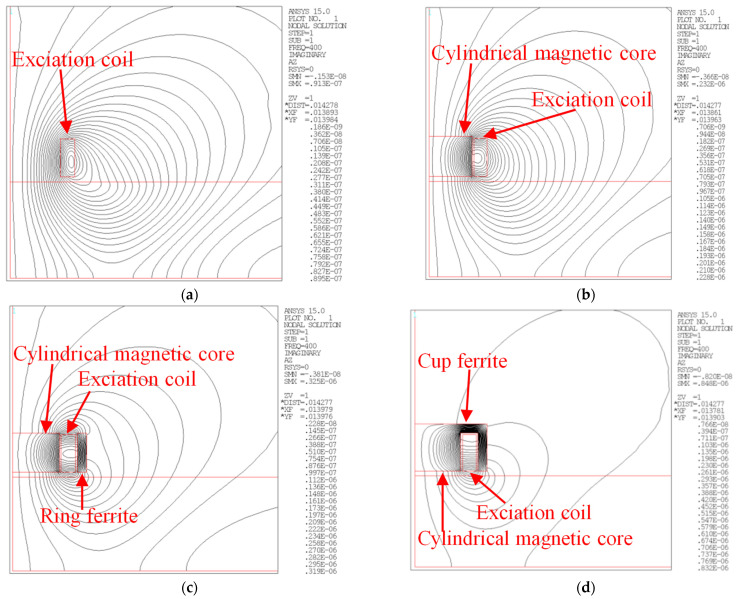
Distribution of magnetic lines of force of different magnetic circuit models. (**a**) Model 1; (**b**) Model 2; (**c**) Model 3; (**d**) Model 4.

In order to compare the magnetization effects of magnetic circuits with different structures, the parameters of the excitation coil remain unchanged, and four simulation models are established, as shown in Figure 6. There is only an excitation coil in Model 1. In model 2, a cylindrical ferrite is added inside the excitation coil. In model 3, an annular ferrite is added outside the excitation coil on the basis of model 2. In model 4, cup-shaped ferrite is added outside the excitation coil on the basis of model 2 to form a connected magnetic circuit.

The magnetic circuit and the excitation coil used in the simulation are all axisymmetric structures, which can be simplified into two-dimensional simulation models. The magnetic flux distribution lines of different magnetic circuit models are calculated, and the simulation results are shown in Figure 4.

By comparing the magnetic field distribution of the four models, it can be seen that when the excitation coil is not equipped with any magnetic conductive structure, the magnetic field generated by the excitation coil in space is relatively divergent. There is a strong direct coupling magnetic field above the tested component, while the indirect coupling magnetic field inside the tested component is so weak that the remote field eddy current effect cannot be realized. By continuously adding a magnetization structure to the excitation coil, the excitation magnetic field is gradually concentrated near the excitation coil, and the internal magnetic field of the measured component is effectively enhanced.

**Figure 7:**
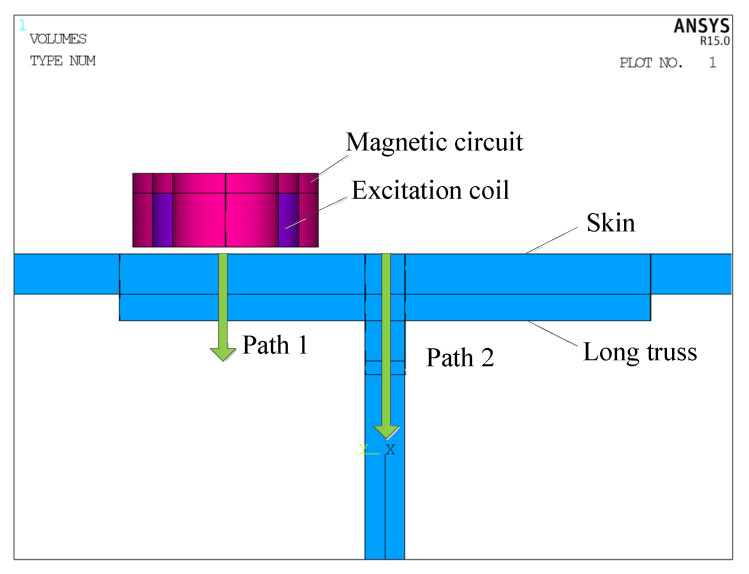
Schematic diagram of magnetic field strength extraction path setting.

**Figure 8:**
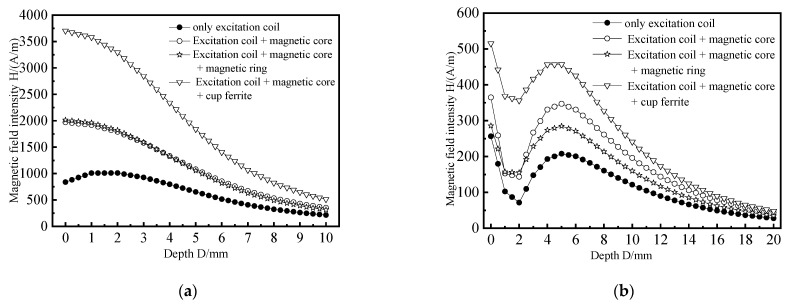
The influence of the magnetic circuit structure on the indirect coupling magnetic field. (**a**) Directly below the excitation coil; (**b**) Web position.

In order to better compare the influence of different magnetic circuit models on the indirect coupling magnetic field, a path is set in the depth direction of the measured component directly below the excitation coil and the depth direction of the web of the long truss structure, as shown in Figure 7. The variation trend of the magnetic field directly below the excitation coil and in the depth direction of the web of the long truss structure are extracted, and the results are shown in Figure 8.

From Figure 8a, it can be seen that when the excitation coil is not installed with any magnetic conducting structure, the magnetic field intensity curve of the coil is monotonically decreasing. The magnetic field intensity of model 1 is 652.2 A/m when the plate thickness is 5 mm. The magnetic intensity of model 2, model 3, and model 4 is 1082.3 A/m, 1054.8 A/m, and 1838.2 A/m, respectively, and the increase ranges are 65.9%, 61.7%, and 181.8%, respectively. It can be seen from Figure 8b that the magnetic field intensity curve of the web of aircraft long truss structure decreases first, then increases, and then decreases slowly. The magnetic field intensity of the excitation coil is 121.5 A/m when the plate thickness is 10 mm. The magnetic intensity of model 2, model 3 and model 4 is 159.8 A/m, 196.0 A/m, and 240.8 A/m, respectively, and the increase ranges are 31.5%, 61.3%, and 98.2%, respectively. By comparison, it can be found that after the excitation coil is equipped with a connected magnetic circuit composed of a cylindrical magnetic core and a cup-shaped ferrite, the internal magnetic field strength of the aircraft long truss components increases the most, and the magnetic field concentration effect of the connected magnetic circuit is the best.

#### 3.1.2. The Influence of Magnetic Circuit Thickness on Indirectly Coupled Magnetic Field

**Figure 9:**
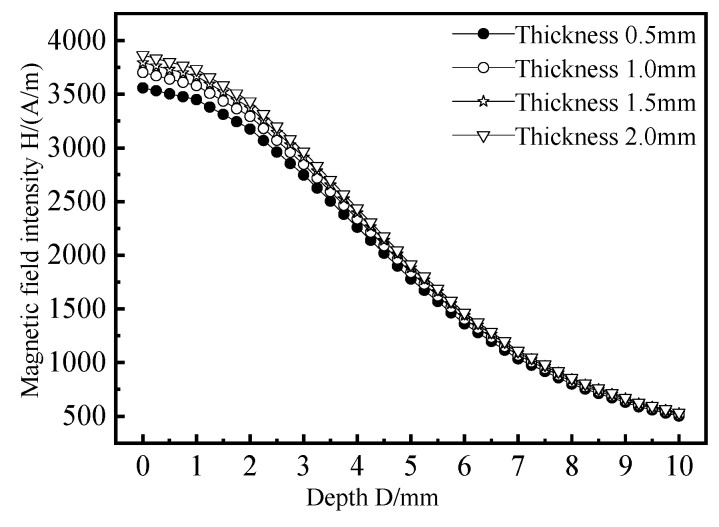
The influence of magnetic circuit thickness on indirectly coupled magnetic field.

The size of the magnetic circuit coupling structure affects the magnetization effect of the entire magnetic circuit. To compare the magnetic field enhancement effect of different thickness magnetic circuits and optimize the magnetic circuit thickness, the excitation coil parameters and the magnetic circuit structure are kept unchanged, and the magnetic circuit thickness T is changed. The simulation results in Figure 9 show the changing trend of the indirect coupling magnetic field in the depth direction of the measured component. It can be seen that the intensity of the magnetic field inside the measured component is further enhanced as the thickness increases. Therefore, it can improve the magnetizing effect of the magnetic circuit by increasing the thickness of the connected magnetic circuit appropriately.

### 3.2. Simulation Optimization of Shielding Suppression Unit

**Figure 10:**
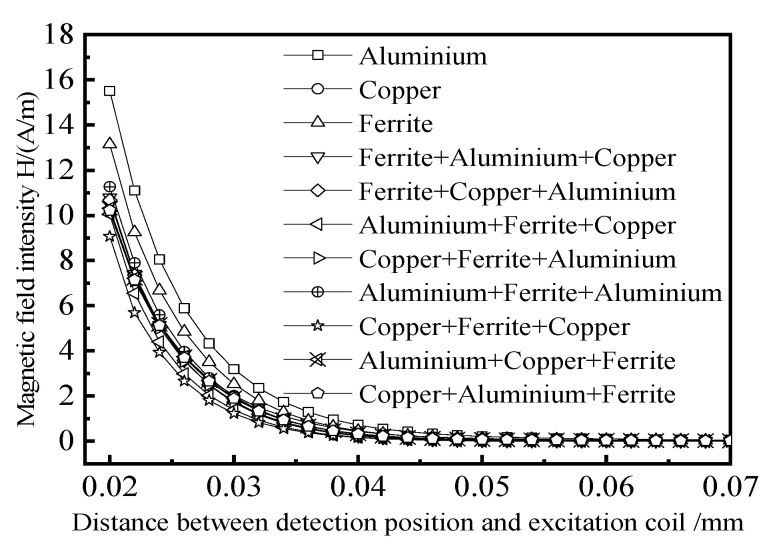
The influence of shielding material on direct coupling magnetic field.

The shielding suppression unit adopts the method of composite shielding of multiple materials. The number of layers is three, and the total thickness is 6 mm, so as to accelerate and weaken the magnetic field energy on the direct coupling path and shorten the distance between the remote field area and the excitation coil. The conductivity and relative permeability of the shielding layer material is set as shown in Table 1. In the simulation, single materials of aluminum, copper, and ferrite and different combinations of the three materials are used to study the shielding damping structure. The thickness of each layer is 2 mm. After the direct coupling energy passes through the shielding damping structure of different materials, the changing trend of the direct coupling magnetic field above the tested component is compared. The simulation results are shown in Figure 10.

It can be seen that when the direct coupling energy passes through the composite shielding damping structure of copper + ferrite + copper, the direct coupling magnetic field above the tested component is the weakest, indicating that the combination of copper + ferrite + copper has the best shielding effect on the direct coupling energy. The shielding effect of aluminum + ferrite + copper combination is the second. The conductivity of the first layer of copper is better than that of aluminum, so the direct magnetic field energy can induce a stronger eddy current in the copper to generate a stronger reverse magnetic field and attenuate the direct coupling energy. The ferrite in the middle layer is a high permeability material, which can accumulate direct coupling energy after passing through the first layer of copper. The copper in the outermost layer attenuates the direct coupling energy accumulated by the ferrite twice. Therefore, the combination of copper + ferrite + copper is selected as the shielding damping structure for further research.

### 3.3. The Influence of Excitation Frequency on Detection Signal

**Figure 11:**
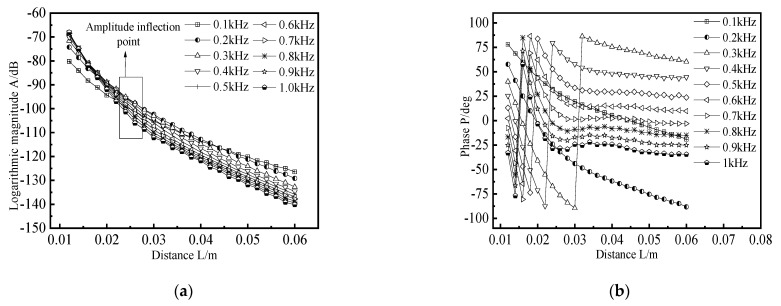
Amplitude–distance curve and phase–distance curve under different excitation frequencies. (**a**) Amplitude–distance curve; (**b**) Phase–distance curve.

The detection frequency of remote field eddy current belongs to the low-frequency band, usually between tens of Hertz and thousands of Hertz. The excitation frequency can affect the distance between the remote field region and the excitation coil, which requires simulation research on the excitation frequency. Based on the existing excitation model of the remote field eddy current probe, the detection coil is added, and the excitation frequency f and the distance L between the excitation coil and the detection coil are changed. The excitation frequency f varies from 100 Hz to 1 kHz. The variation range of L is 12 mm to 60 mm, step 2 mm. The amplitude and phase of the induced voltage at different positions of the detection coil are obtained. The logarithmic amplitude–distance and phase–distance curves are drawn, as shown in Figure 11.

It can be seen from Figure 11a that when the detection coil is near 25 mm from the excitation coil, an inflection point appears in the logarithmic amplitude–distance characteristic curve. Additionally, the higher the excitation frequency, the more obvious the inflection point. As can be seen from Figure 11b, when the excitation signal frequency f is less than 200 Hz, the phase change is not obvious. When the frequency f is greater than 200 Hz, the phase distance characteristic curve changes suddenly in the range of 20 mm to 30 mm. With the increase of frequency, the phase change gradually approaches the excitation coil, which conforms to the characteristics of “amplitude inflection point” and “phase hit” of remote field eddy current. The higher the excitation frequency, the smaller the distance between the position of the remote field region and the excitation coil.

### 3.4. The Influence of Excitation/Detection Coil Spacing on Detection Sensitivity

**Figure 12:**
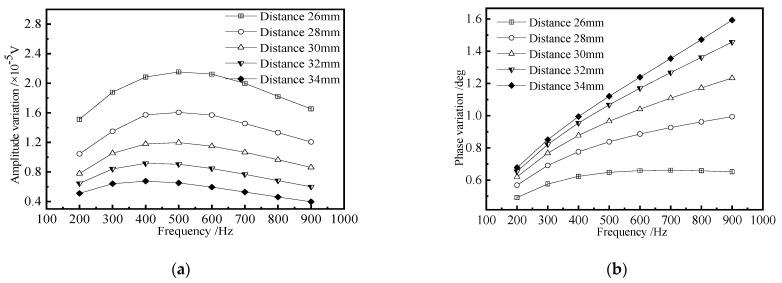
The influence of the excitation/detection coil spacing on the detection signal. (**a**) Detection signal amplitude; (**b**) Detection signal phase.

In traditional remote field eddy current testing, there is a near-field region, a transition region, and a remote field region. If the excitation coil and the detection coil are too close, the near-field energy is directly coupled to the detection coil, and the remote field eddy current cannot be used for detection. If they are too far apart, the induced voltage of the detection coil is too weak. Fukutomi [23] found that in the remote field eddy current testing of non-ferromagnetic steam generator tubes, when the detection coil is located in the transition zone, the detection signal of the RFECT probe for outer wall defects is higher than that of inner wall defects with the same size. In order to study the influence of excitation/detection coil spacing on detection sensitivity and determine the optimal distance L between excitation coil and detection coil, a rectangular groove is added to the long truss web structure to simulate crack-like defects. The excitation frequency is changed to scan the same defect. The length × width × height of the defect is 20 × 1 × 3 mm, and the burial depth is 8 mm. The excitation/detection coil distance L varies between 26 mm and 34 mm, and the step is 2 mm. The amplitude change and phase change of the detection signal are obtained by subtracting the detection signal without defect from the detection signal with a defect. The simulation results are shown in Figure 12.

It can be seen from Figure 12 that when the distance L between the excitation coil and the detection coil is constant, the amplitude change curve first slowly rises and then slowly decreases with the increase of frequency, and the phase increases monotonically with the increase of frequency. At the same excitation frequency f, the amplitude change of the detection signal decreases with the increase of distance L, and the phase change increases with the increase of distance L. When the distance L is 26 mm, the amplitude change caused by defects is the largest, but the phase change is the smallest, which is not conducive to the distinction between defect signal and noise signal in actual detection. When the distance L is 34 mm, the amplitude change caused by defects is the smallest, but the phase change is the largest, which is easy to cause missed detection of defects. Considering the influence of distance on the amplitude and phase of defect signal, 30 mm is selected as the distance between the excitation coil and detection coil, which not only ensures that the defect signal has a certain amplitude but also makes the defect signal have a large phase change. In the later simulation calculation, the detection coil is placed 30 mm away from the excitation coil, and the excitation frequency is selected as 0.5 kHz for further research.

### 3.5. Influence of Excitation Coil Position on Detection Sensitivity

**Figure 13:**
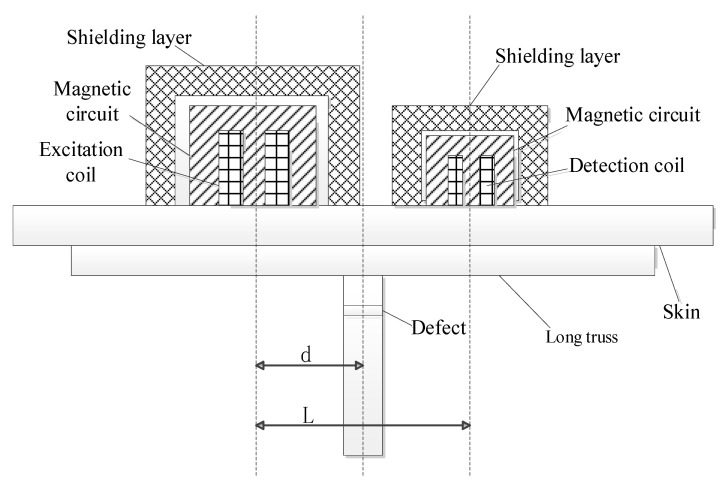
Schematic diagram of remote field eddy current testing method for aircraft long truss components.

**Figure 14:**
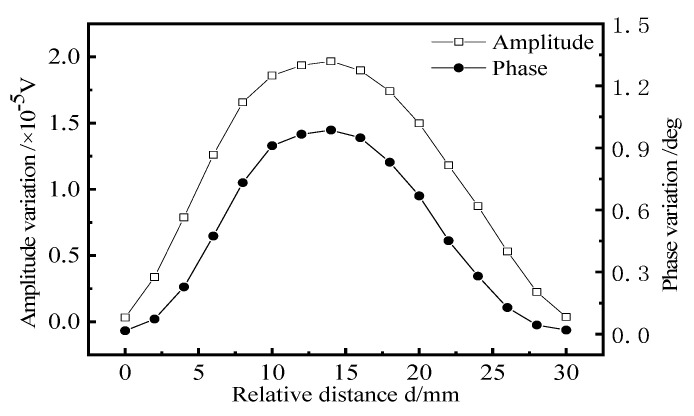
Influence of excitation coil position on detection signal.

When the remote field eddy current probe is used to detect the aircraft long truss components, the excitation coil is located on the side of the web. Figure 13 shows the schematic diagram of remote field eddy current testing method for aircraft long truss components. The relative position of the excitation coil and web directly affects the magnetic field strength of the defect position. Therefore, it is necessary to study the placement position of the excitation coil to find the best placement position for the remote field eddy current probe. In the simulation, the distance d between the center of the excitation coil and the center of the web changes from 0 to 30 mm in steps of 2 mm. At 0 mm, the excitation coil is located directly above the web, changing the position of the excitation to scan the same defect. The length × width × height of the defect is 20 × 1 × 3 mm, and the burial depth is 8 mm. The amplitude change and phase change of the defect signal at different positions of the excitation coil are obtained. The results are shown in Figure 14.

As can be seen from Figure 14, with the increase in the distance between the excitation coil and the web, the amplitude and phase change curve of the defect signal shows a trend of first rising and then falling, reaching the maximum value when d is 1/2 L. At this time, the excitation coil and the detection coil span the web and are symmetrical about the web. When the excitation coil is located at this position, the probe has the highest sensitivity to detect crack-like defects. When d is 0 mm or 30 mm, the excitation coil and detection coil are located directly above the web, respectively. At this time, the amplitude change and phase change of the defect signal are basically zero, indicating that the detection sensitivity of these two detection methods is the lowest.

## 4. Simulation of Crack-Like Defect Detection of Aircraft Long Truss Components

**Figure 15:**
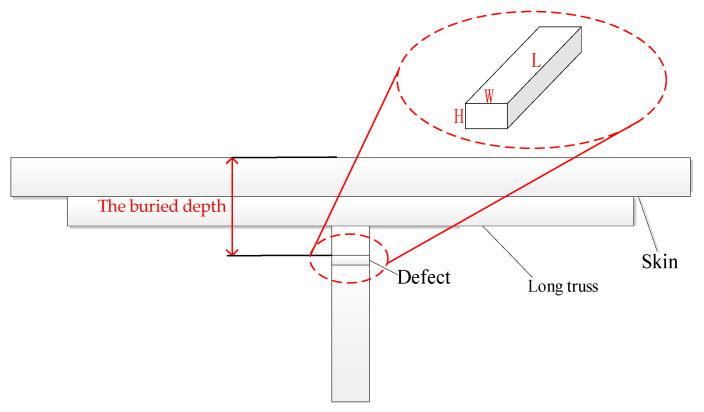
Crack schematic.

How to quantitatively measure the length, buried depth, and depth of defects is an important research subject for non-destructive testing. In the simulation of crack-like defect detection of aircraft long truss members, by changing the crack-like defect size parameters, the effects of defect length and buried depth on the detection signal are studied to find the relevant quantitative law. The schematic diagram of cracks is shown in Figure 15. In order to facilitate analysis, the simulation data are normalized based on the induced voltage at the defect-free position. The excitation frequency f is set to 500 Hz, and the excitation current I is set to 100 mA.

### 4.1. Influence of Defect Length on Detection Signal

**Figure 16:**
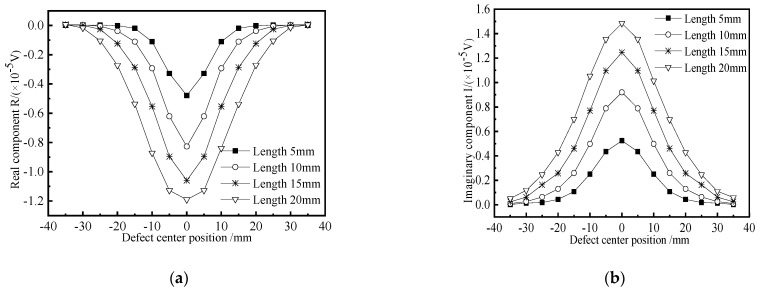
Crack defect detection signals of different lengths. (**a**) Real component; (**b**) Imaginary component.

In order to study the influence of crack-like defect length on the detection signal, four defects with different lengths are simulated and established for scanning. The buried depth of the defect is 8 mm, the width is 1 mm, the depth is 3 mm, and the lengths are 5 mm, 10 mm, 15 mm, and 20 mm, respectively. The change curves of the real part and the imaginary part of the defect signal are obtained. The simulation results are shown in Figure 16. It can be seen from the figure that the real part and imaginary part curves of the detection signal show a trend of rising first and then falling and show very good symmetry with respect to the other scanning positions on both sides of the crack-like defect. When the probe scans the defect center, the real part component of the detection coil voltage reaches the negative maximum, and the imaginary part component reaches the positive maximum. The longer the defect, the greater the peak value of the curve. The existence of defects disturbs the flow of induced eddy current in the tested component, resulting in the change of coil impedance. With the increase of the defect length, the maximum value of the detection signal gradually increases, but the increasing amplitude decreases because the magnetic field energy distribution is concentrated after the probe increases the magnetic focusing and shielding structure. Although the length of the defect increases exponentially, the contribution value of each part of the defect to the magnetic field disturbance in the length direction is different.

### 4.2. Influence of Defect Buried Depth on Detection Signal

**Figure 17:**
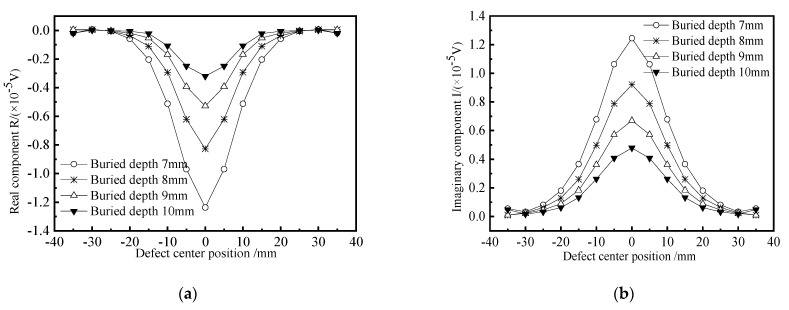
Crack defect detection signals at different buried depths. (**a**) Real component; (**b**) Imaginary component.

In order to study the influence of the buried depth of crack-like defects on the detection signal, four kinds of defects with different buried depths are simulated and established for scanning. The length × width × height of defects is 10 × 1 × 3 mm, and the buried depths are 7 mm, 8 mm, 9 mm, and 10 mm, respectively. The change curves of the real part and the imaginary part of the defect signal are obtained. The simulation results are shown in Figure 17. It can be seen that when the probe scans the defect center, the real part component of the detection coil voltage has a negative peak, and the imaginary part component has a positive peak. The real part and imaginary part curves of the detection signal show good symmetry with respect to the other scanning positions on both sides of the crack-like defect. With the increase of the buried depth of the defect, the peak value of the detection signal decreases gradually, but the decreasing amplitude decreases. Because with the increase of the depth, the excitation magnetic field is weakened due to the influence of the skin effect. As a result, the disturbance of defects with the same volume equivalent to the magnetic field will be reduced.

## 5. Test Verification

**Figure 18:**
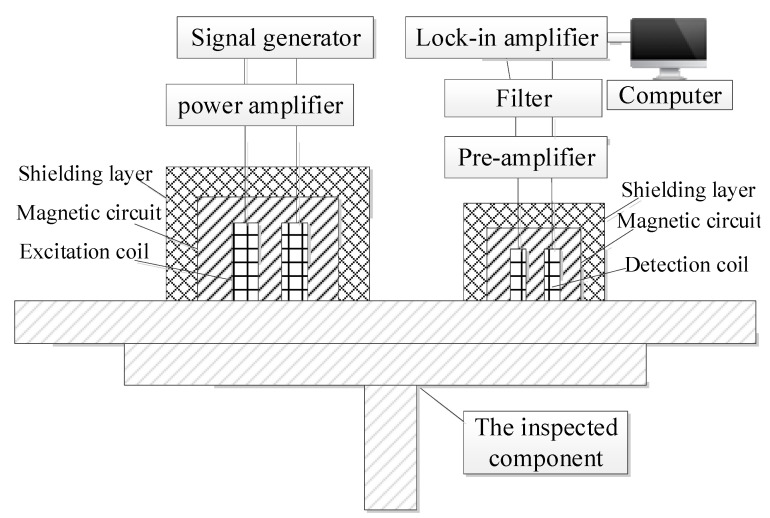
Block diagram of remote field eddy current testing system for aircraft long truss structure.

**Figure 19:**
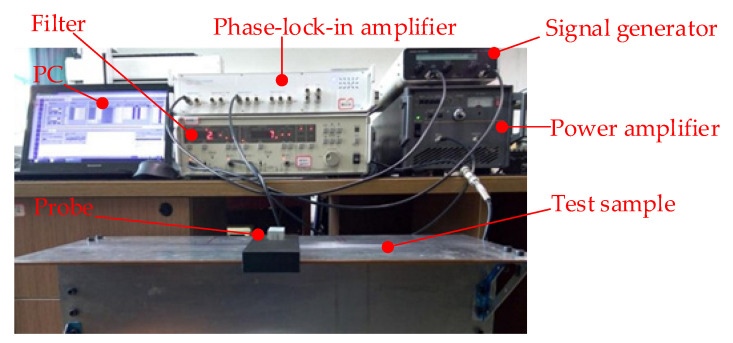
Physical drawing of PRFECT system for aircraft long truss structure.

**Figure 20:**
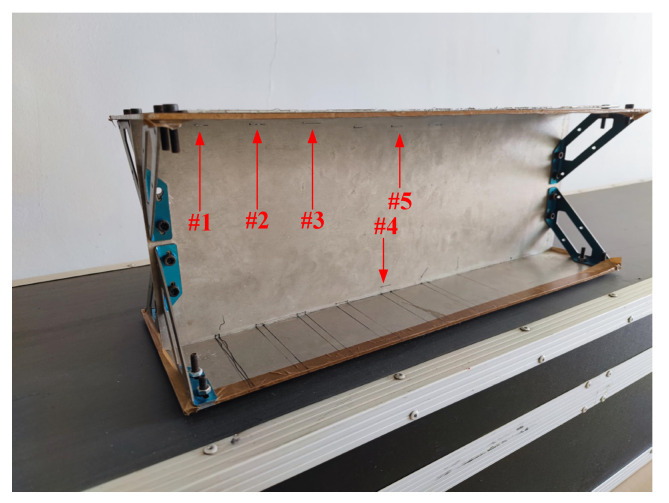
Test sample. (#1) 5 × 0.2 × 3 mm (Buried depth: 6 mm); (#2) 10 × 0.2 × 3 mm (Buried depth: 6 mm); (#3) 15 × 0.2 × 3 mm (Buried depth: 6 mm); (#4) 10 × 0.2 × 3 mm (Buried depth: 8 mm); (#5) 10 × 0.2 × 3 mm (Buried depth: 10 mm).

In order to verify the detection effect of the model on the defects of the long truss structure, the probe is made according to the model design, and a test system is built for verification. The detection test system is shown in Figure 18 and Figure 19. The test sample is shown in Figure 20. The test system includes a signal generator, a power amplifier, a low-frequency pre-amplifier, a phase-lock-in amplifier, and a computer. The signal generator generates an excitation signal, which is amplified by the power amplifier and loaded into the excitation coil to generate an excitation magnetic field. The generated induction signal is picked up by the probe detection coil and amplified by the low-frequency pre-amplifier. The amplified detection signal is filtered out by the filter. Finally, the data is collected by the lock-in amplifier and stored in the computer for corresponding processing and analysis.

### 5.1. Detection of Defects with Different Lengths

**Figure 21:**
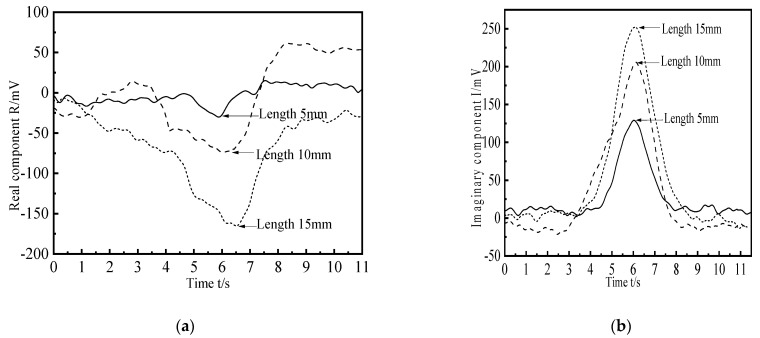
Real and imaginary components of crack-like defect detection signals with different lengths. (**a**) Real component; (**b**) Imaginary component.

The excitation frequency *f* is set to 500 Hz, and the excitation current *I* is set to 100 mA to detect crack-like defects with different lengths. Additionally, the influence of defect length on eddy current signal is studied. The defect is a through crack, with a buried depth of 6 mm, a width of 0.2 mm, and a length of 5 mm, 10 mm and 15 mm, respectively, as shown in defects #1, #2, and #3 in Figure 20. The real and imaginary components of the detection signal are shown in Figure 21. It can be seen that when the probe scans directly above the defect, both the real and imaginary parts of the detection coil voltage peak when the probe is scanned directly over the defect. The peak value of the detection signal increases with the increase of the defect length. When t varies from 1 s to 6 s, the real part of the defect detection voltage with a length of 5 mm increases from −13.7 mV to −29.4 mV, an increase of 1.15 times, and the imaginary part increases from 11.7 mV to 128.9 mV, an increase of 10.02 times. When the length is 10 mm and 15 mm, the real component increases by 1.46 times and 8.16 times, respectively, and the imaginary component increases by 14.67 times and 44.74 times, respectively. The increased amplitude of the imaginary component of the defect signal is greater than that of the real component.

### 5.2. Detection of Defects with Different Buried Depths

**Figure 22:**
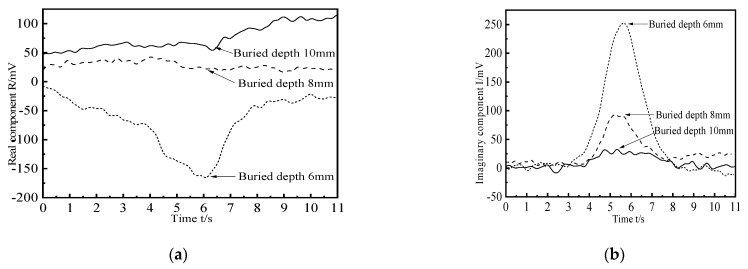
Real and imaginary components of crack-like defect detection signals with different buried depths. (**a**) Real component; (**b**) Imaginary component.

Crack-like defects with different buried depths are detected to study the influence of defect buried depth on the detection signal. The crack-like defect penetrates through the 3 mm thick aluminum plate, with a length of 10 mm, a width of 0.2 mm, and a buried depth of 6 mm, 8 mm, and 10 mm, respectively, as shown in defects #2, #4, and #5 in Figure 20. The defect is scanned by a uniform sliding probe. The results of the real and imaginary components of the detection signal are shown in Figure 22. As can be seen from Figure 22a, when the buried depth of the defect is equal to 6 mm, the real part of the detection voltage has a negative peak. When the buried depth of the defect is greater than 6 mm, the change of the real part component of the detection signal is not obvious, and there is no defect signal feature. As can be seen from Figure 22b, when the buried depth of the defect is equal to 10 mm, the imaginary component of the detection signal still changes obviously, and the characteristics of the defect signal are obvious. With the increase of the buried depth of the defect, the peak value of the imaginary component decreases.

## 6. Conclusions

Taking the aircraft long truss structure as the research object, this paper established a three-dimensional finite element simulation model of remote field eddy current testing and analyzed the distribution characteristics of the electromagnetic field. The remote field eddy current testing probe was improved from two aspects of excitation magnetic field enhancement and direct coupling energy suppression. The magnetic circuit, shielding layer, excitation/detection coil spacing, and probe placement were simulated and analyzed. The remote field eddy current effect was realized in the aircraft long truss structure.

(1) The magnetic core + cup ferrite structure was selected to form a connected magnetic circuit, which had the best effect on magnetic field aggregation. By increasing the thickness of the magnetic circuit, the magnetic focusing effect of the magnetic circuit was improved.
(2) Copper + ferrite + copper was selected as the shielding layer material to form a composite shielding layer, which had a good shielding effect on the direct coupling energy.
(3) On the premise of ensuring that the phase of the defect signal and no defect signal could be clearly distinguished, the detection sensitivity of the probe was effectively improved by shortening the spacing between excitation/detection coils. When the excitation coil and detection coil were placed symmetrically about the aircraft truss web, the detection sensitivity of the probe was the highest. Additionally, the higher the excitation frequency, the smaller the distance between the position of the remote field region and the excitation coil.
(4) Simulation and experiments verified that with the increase of defect length, the maximum value of the detection signal gradually increased, but the increased amplitude decreased. With the increase of defect buried depth, the peak value of the detection signal gradually decreased, but the decreased amplitude decreased. The final designed PRFECT probe detected the crack defects in the aircraft long truss structure with a buried depth of less than 10 mm. In defect characterization, the imaginary component was better than the real component.

## Figures and Tables

**Table 1:** Setting of conductivity and relative permeability of shielding material.

Material	Aluminium	Copper	Ferrite
Conductivity σ	25.5 × 10^6^	58.8 × 10^6^	0
Relative permeability $\mu_{r}$	1	1	1000

## Data Availability

Not applicable.
